# Exploring the links between swimming performance, glucocorticoid profiles, behavioral types and cardiac morphology in migrating Atlantic salmon *(Salmo salar)* smolts

**DOI:** 10.1038/s41598-026-35402-y

**Published:** 2026-01-16

**Authors:** Erik Höglund, Kurt Johansen, Silje Marie Ulset, Elise T. Sannes, Tormod Haraldstad, Ida B. Johansen, Michael Frisk, Marco A. Vindas, Marta Moyano

**Affiliations:** 1 Norwegian Institute for Water Research (NIVA), Økernveien 94, 0579 Oslo, Norway; 2 Center for Coastal Research, University of Agder, Universitetsveien 25, 4604 Kristiansand, Norway; 3 NORCE, Universitetsveien 19, 4630 Kristiansand, Norway; 4 Department of Preclinical Sciences and Pathology, Faculty of Veterinary Medicine, Norwegian University of Life Sciences, Ås, Norway; 5 Insitute for Experimental Medical research, University of Oslo and Oslo University Hospital Ullevål, Oslo, Norway

**Keywords:** Ecology, Evolution, Neuroscience, Physiology

## Abstract

It has been become increasingly clear that swimming performance is crucial for the migratory success of wild Atlantic salmon (*Salmo salar*) smolts, yet the associated traits remain largely unexplored. In this study, smolts were caught during their downstream migration to the sea and were categorized as strong or poor swimmers based on their time to fatigue in a swimming test. Behavioral profiles were characterized through a confinement and novelty test, and relationships to cardiac morphology and glucocorticoid profiles were investigated. Strong swimmers increased activity over time in the confinement test, while poor swimmers showed the opposite trend, indicating differences in boldness. There were no differences in plasma cortisol in groups of fish with contrasting swimming performance. Still, strong swimmers had higher cortisone levels 24 h after the swim test, suggesting a protective mechanism against elevated cortisol in this group. No differences in heart morphology were found between the groups. In addition, acoustic telemetry revealed a trend toward lower survival rates for strong swimmers in predator-rich environments. Overall, our results highlight a link between glucocorticoid profiles, boldness and swimming capacity in Atlantic salmon smolts. Further studies are needed to confirm the observed survival patterns and to better understand how predator pressure may shape the fitness consequences of these traits in migrating smolts.

**Supplementary Information:**

The online version contains supplementary material available at 10.1038/s41598-026-35402-y.

## Introduction

It has become increasingly clear that swimming performance plays a crucial role in the migratory success of wild Atlantic salmon (*Salmo salar*) from natal rivers to ocean feeding grounds^1^. Despite its critical importance, the physiological factors and other traits associated with swimming capacity in wild salmon remain largely unexplored. Understanding these traits can provide valuable insights into the determinants of fitness and survival in this species.

Previous studies have linked swimming performance to cardiac morphology, particularly in farmed populations of rainbow trout (*Oncorhynchus mykiss*)^2^. Farming conditions are associated with a rounded ventricular phenotype, which impairs swimming ability compared to individuals with more elongated and pyramid-shaped ventricles. This finding is particularly relevant, as there is substantial variation in heart shape among Atlantic salmon smolts from natural populations^3^. However, it remains unknown whether this cardiac variation has a functional impact on the swimming performance of migrating wild salmon. Physiological factors influencing heart size and tissue composition may also impact cardiovascular and physical performance. For example, elevated cortisol levels are associated with hypertrophic cardiac growth, which has been linked to impaired swimming performance in rainbow trout^4^. Of note, cortisol levels peak during the smoltification process^5^ and the magnitude of such neuroendocrine responses is subject to large individual variation in salmonids, including Atlantic salmon^6,7^. Nonetheless, the potential relationship between cortisol levels, cardiac morphology, and swimming capacity in wild Atlantic salmon smolts remains to be investigated.

Consistent individual differences in behavior, or behavioral types (BT), often correlate with aerobic performance. For example, individual variation in the bold-shy continuum has been linked to key physiological indicators like standard metabolic rate (SMR), maximum metabolic rate, and aerobic scope, which are closely tied to swimming capacity and cardiorespiratory performance^8–11^. Moreover, BTs, have been shown to be reflected in the locomotor activity and neuroendocrine profiles of animals^12,13^. Alluding to this, studies in salmonid fishes have shown that locomotor activity is associated with boldness and cortisol responsiveness to stress^13,14^. Still, the relationship between aerobic performance, BT, and glucocorticoid profiles during smoltification—an ontogenetic shift when cortisol levels peak and seaward migrating young salmon are imprinted with cues for returning to spawning grounds^15^—remains, to our knowledge, unknown.

As described above, fish studies show that aerobic and swimming performance are strongly associated with an individual’s BT. Furthermore, studies in sockeye salmon (Oncorhynchus nerka) demonstrate that Intrinsic differences in aerobic performance contribute to variability in migratory success^16^. Still, whether these factors are related to survival during the critical phase of downstream migration in Atlantic salmon remains unexplored.

This study aimed to investigate the relationships between behavior, glucocorticoid profiles, heart morphology, and swimming performance in migrating Atlantic salmon smolts. To achieve this, smolts were captured using a wolf-trap during their downstream migration in the Nidelva River, southern Norway, and categorized as strong or poor swimmers based on a standardized swimming performance test^2^. A subset of individuals was sampled for analyses of heart morphology and plasma glucocorticoid levels. Additionally, activity levels in response to novelty and confinement in strong and poor swimmers were examined in a controlled tank test^17^. Finally, acoustic telemetry was employed to study the behavior of a subset of strong and poor swimmers during their downstream migration. We hypothesized that swimming performance in salmon is linked to their cardiac ventricular phenotype, glucocorticoid profile, behavioral traits, or a combination of these factors, and that this would impact migratory success and survival.

## Material and methods

### Experimental animals

Salmon smolts were caught using a wolf-trap (Philip Wolf, 1951) at the Rygene hydroelectric power (HEP) station in the Nidelva river, southern Norway (59.41540°N, 8.74343°E), in May 2021.

### Ethics declaration

The experiment was conducted in accordance with the Guidelines of the European Union Council (86/609/EU) and Norwegian legislation for the use of laboratory animals. The experimental protocol was approved by the ethics committee of the Norwegian food safety authority (permit number 23023). Furthermore, the methods reported in accordance with ARRIVE guidelines (https://arriveguidelines.org).

### Swimming test challenge

First, a swimming test was done with groups of smolts (> 50 individuals) to sort them into strong and poor swimmers based on the time to exhaustion. The test was performed in a raceway tank (2.5 m inner diameter) using a modified protocol based on the study performed by Claireaux et al. (2005). Water in the tank was pumped from the river for > 1 h before the test started and then throughout the test. Current in the tank was generated by two propellers (Biltema 25–2302, Norway) that were separated from the main tank by a mesh. Fish collected from the trap were introduced in the tank and let to acclimate for 1.5 h. Once the swimming test started, the current in the tank was raised at approx. 0.2 m s^-1^ and then increased stepwise to approximately 1 m s^-1^ in 5 min, corresponding to 6.3–9.1 body length s^-1^ depending on fish size. This current remained throughout the test. Water current measured regularly using a vane probe (md20 5329, Höntzsch Flowtherm Germany). During the trials, we observed that some fish tended to aggregate in low-flow areas, particularly near the inner wall of the flume where turbulence was higher. To ensure consistent engagement with the flow and to promote exhaustion, these individuals were gently encouraged to re-enter the main current using a dip net. Smolts were removed from the tank when they were considered to be exhausted, i.e. laying against the back mesh and were unresponsive to netting. The first 20% exhausted smolts were considered poor swimmers, and the last 20% swimming smolts were considered strong swimmers. The remaining 60% were considered intermediate swimmers and were omitted from further testing. Tests ended when there were only strong swimmers left. Note that the 20% cutoff differed slightly among tests, being between 18 and 21% (see Table 1). Only in Test #1, the number of fish classified as poor swimmers was 32% because several fish were exhausted at the same time. The total duration of the test ranged between 42 and 90 min.

**Table 1:** Number of fish classified as strong or poor swimmers upon reaching exhaustion during a group swimming performance test. Four bouts were conducted in total. After classification, the fish were behaviorally profiled, sampled for tissue and blood plasma, or their migratory behavior were studied using acoustic telemetry.

Test #	Test date	Temp start (°C)	Temp end (°C)	Total fish (n)	Group size (n)		Behavior profil. (n)		Tissue and plasm. (n)		Telemetry (n)	
					Strong	Poor	Strong	Poor	Strong	Poor	Strong	Poor
1	12.05.2021	7.1	7.1	52	10	17	2	6	8	11	–	–
2	13.05.2021	7.0	7.3	66	14	12	–	–	8	10	–	–
3	18.05.2021	9.3	9.3	95	18	20	9	7	–	–	7	7
4	18.05.2021	9.3	9.5	99	21	20	13	10	–	–	8	8

A total of four bouts were performed (Table 1). Temperature was stable during the tests (± 0.3 °C), but it slightly increased throughout the experimental period (Table 1).

After the swimming test, all fish were measured (length and wet weight) and were either sampled (see below for further details) or subjected to the behavioral confinement/novelty tank test. The strong and weak swimmers were placed in separate chambers in a holding tank to recover overnight before being sampled, behaviorally profiled or inserted with acoustic tags the next day.

### Plasma and tissue sampling

A total of 31 wild salmon were sampled (17 strong and 14 poor swimmers) for plasma glucocorticoids and cardiac morphology. Fish were lightly sedated on an MS222 (Finquel vet, Western Chemical Inc, Washington DC, USA) bath (0.1 g/L) before they were measured (fork length with a calliper) and weighed (wet weight, Soehnle, Purista scale, precision 0.2 mg/L). Then, fish were euthanized with a lethal dose of buffered MS-222 (2 g/L). Once fished showed no signs of life (within 30 s) a blood sample was taken from the caudal vein using a 23G, 1 ml syringe containing ethylene diamine tetra acetic acid (EDTA) as an anticoagulant. Samples were kept on ice until they were centrifuged for 10 min at 700 g in a portable mini centrifuge at outdoor temperatures. The supernatant was then immediately frozen in Eppendorf tubes on dry ice and stored at − 80 °C for later analysis. The hearts were then removed, rinsed in PBS and then transferred to 20 mM KCL solution to arrest the heart in diastole. Once hearts ceased beating, they were weighed and photographed as described below. For a subset of hearts (n = 10), the atrium and bulbus were removed to assess ventricular mass.

### Imaging and analysis of cardiac morphology

Freshly excised hearts were photographed from standardized left lateral and ventrodorsal projections inside a Styrofoam box (internal dimensions: H 24 cm × W 21 cm × L 27 cm) illuminated by an internal LED light (Northlight LED lamp, Art. No. 36–6465). Images were captured using a Canon EOS 4000D camera equipped with an EF-S 18–55 III lens, mounted directly on the Styrofoam box to ensure consistent positioning and lighting conditions across all samples.

Heart measurements were calculated and analyzed following the methods described in Engdal et al.^3^. From the ventrodorsal projection, the ventricular height-to-width ratio was determined by dividing the ventricle length (apex to bulbus) by its maximum width. In the left lateral projection, ventricular symmetry was assessed by measuring the angle between the ventricle’s vertical axis and the line running from the ventriculobulbar groove to the left dorsal ventricular apex. In the same view, the alignment of the bulbus arteriosus was quantified by the angle between its horizontal axis and the ventricular vertical axis. Additionally, the bulbus-to-ventricle width ratio was calculated from the ventrodorsal view by dividing the maximum bulbus width by the maximum ventricular width.

All measurements were performed using Fiji software^18^.

### Plasma cortisol and cortisone

Plasma cortisol and cortisone were quantified reverse phase-liquid chromatography coupled to tandem mass spectrometry (LC–MS/MS) analysis as described in S1 (for retention times and analytical thresholds see Tables S1 and S2, respectively).

### Behavioral profiling

A subsample of strong and poor swimmers was behaviorally profiled by their locomotor response to being inserted into an unfamiliar aquarium (25 L × 15W × 20 D cm). This confinement/novel tank test followed the methods from Haraldstad et al.^17^ Briefly, seven aquariums (were placed on a UV light board. Aquariums were separated by a wood wall to avoid visual contact between fish. Water in the tanks was constantly aerated with an air stone. Smolts were introduced into the tanks and fish behavior was recorded in dim light using cameras with UV-filters for 20 min. Locomotor activity was analyzed in the recorded videos using EthoVision XT (Noldus, Version 11). This confinement/novel tank test was performed the morning after the swimming test was conducted (20–22 h post-swimming test) (Table 1).During the time between the swim test and the behavioral test, strong and poor swimmers were kept in separate compartments within a holding tank.

### Behavior and survival during migration

The morning after the swimming test (20–22 h post-test), 30 smolts (n = 15 per phenotype) were anesthetized with MS222 (tricaine methanesulfonate, 2 mg/L) before being tagged internally with acoustic tags (6.3 × 14.5 mm, 1.2 g in air, LP6 Thelma Biotel). The tags were inserted into a 4–5 mm incision made ventrally between the posterior tip of the pectoral fin and the anterior point of the pelvic girdle. After the tag was inserted, the incision was closed with 2 stitches. After tagging, the fish were kept in perforated cages for 12 h to recover and then released into the river downstream of Rygene HEP. Seven smolts died during recovery, 2 poor swimmers and 5 strong swimmers. The downstream movements of smolts were monitored by ten acoustic listening stations (TBR-700), placed along the six-kilometer river stretch to the river mouth.

### Data analysis

Differences in fish size (length and weight), cardiac morphology and plasma cortisol and cortisone were tested between the strong and poor swimmers. Data were checked for normality and homogeneity of variance visually and running Shapiro and Bartletts test. Differences were then tested with a Student’s t-test. When data were not normally distributed (length, weight), a Wilcoxon test was used to test differences among type of swimmer.

A linear mixed-effects model was used to investigate the effect of experimental time and type of swimmer and trial on fish activity, including individual fish as a random effect. The restricted maximum likelihood (REML) method for parameter estimation. Locomotor activity was log10 transformed. Normality of residuals was checked visually with a Q-Q plot and homogeneity of variance with residual vs fitted plots. All analyses were conducted at a significance level of α = 0.05 with R^19^ version 4.2.2.

River migration survival probability and migration time were estimated using generalized linear models (GLMs) in R^19^ version 4.2.2. Candidate models were fitted to test whether swim type (strong/poor) had a significant effect on the response variables. A logit link function was used to model the binomial response (0 = not detected; 1 = detected at the river mouth receiver), while a Gaussian distribution with an identity link was applied to the migration time data. Model diagnostics, including residual plots, were examined to assess the assumptions of normality and homoscedasticity.

## Results

### Experimental fish

**Fig. 1:**
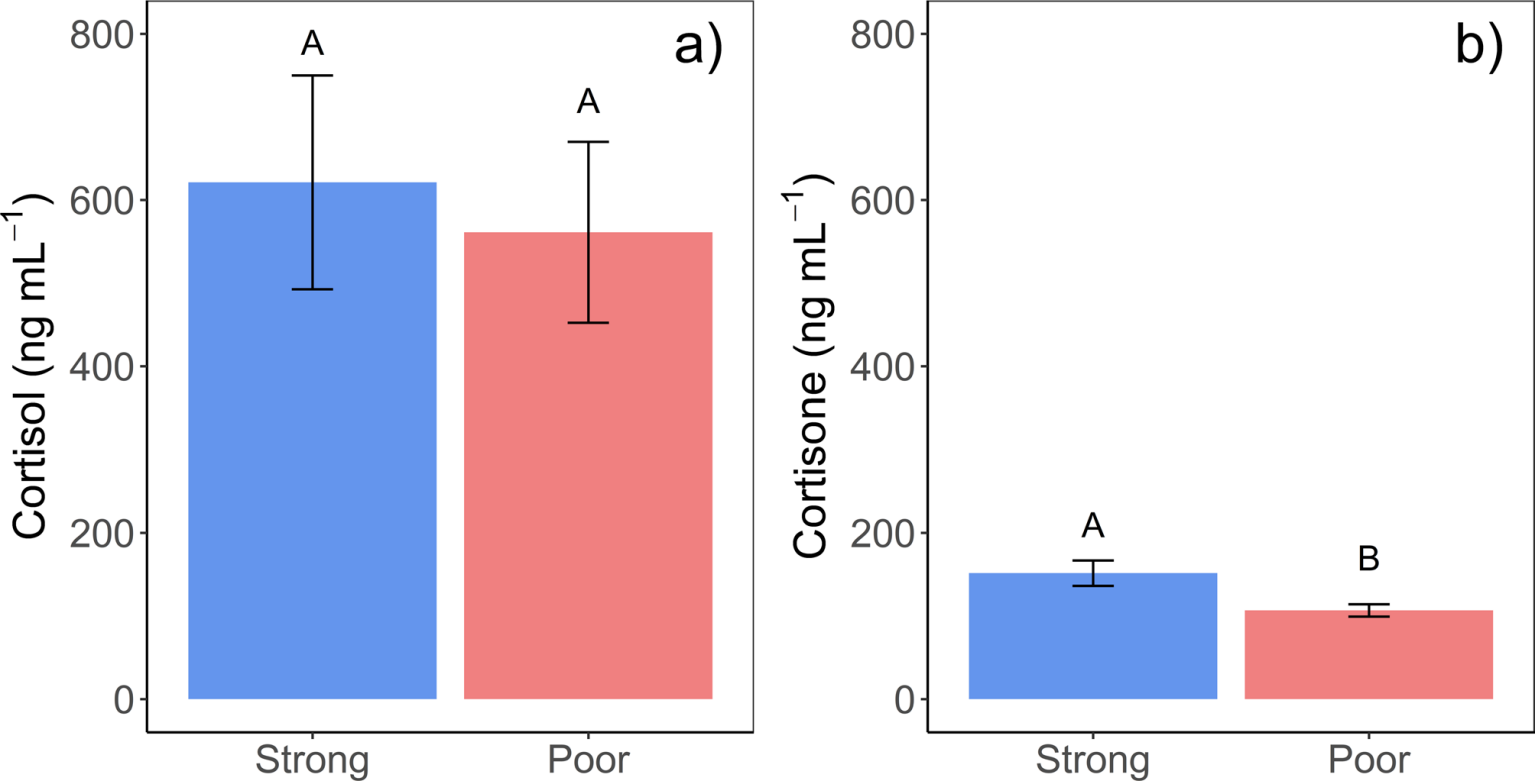
Plasma cortisol and cortisone response to a swim test where fish classified as strong or poor swimmers by the time they reached exhaustion. Average concentrations are shown and error bars indicate standard error (SE). Different letters indicate a significant difference (*P* < 0.05) between strong and poor swimmers.

Strong and poor swimmers had average standard lengths of 13.5 ± 0.22 cm and 13.2 ± 0.16 cm, and wet weights of 22.4 ± 1.04 g and 20 ± 0.73 g (mean ± S.E.M.), respectively (Fig. 1). There were no significant differences between strong and poor swimmers in standard length (W = 1212.5, *p* = 0.400, n = 93) or wet weight (W = 1285, *p* = 0.17, n = 94).

### Heart morphology

There were no significant (*P* < 0.05) differences between strong and poor swimmers in any of the cardiac morphology measurements (Tables 2, S3).

**Table 2:** Cardiac morphology parameters in salmon smolts classified as strong and poor swimmers.

Cardiac morphology	Strong swimmers	Poor swimmers
Cardiosomatic index (CSI)	0.0022 ± 0.00010 (16)	0.0023 ± 0.00006 (21)
Relative ventricular mass (RVM)	0.0016 ± 0.00007 (10)	0.0016 ± 0.00006 (14)
Ventricular height: width ratio	1.1 ± 0.032 (16)	1.1 ± 0.025 (21)
Bulbus width: ventricular width ratio	0.41 ± 0.0090 (16)	0.40 ± 0.0062 (21)
Ventricular bulbus angle	30 ± 2.7 (15)	28 ± 3.4 (21)
Ventricular asymmetry angle	82 ± 2.0 (15)	82 ± 1.8 (21)

Values are mean ± S.E.M (n). Differences were not significant between groups for any of the variables (t-test, *p* > 0.05, Table S3).

### Plasma cortisol and cortisone

Strong swimmers had significantly higher plasma levels of cortisone 24 h after being exposed to the swim test (t =  − 2.6, df = 16.063, *p*-value = 0.018). However, there was no significant difference between strong and poor swimmers in the cortisol following this test (t =  − 0.36, df = 22, *p*-value = 0.72; Fig. 1).

### Behavior profiling

**Fig. 2:**
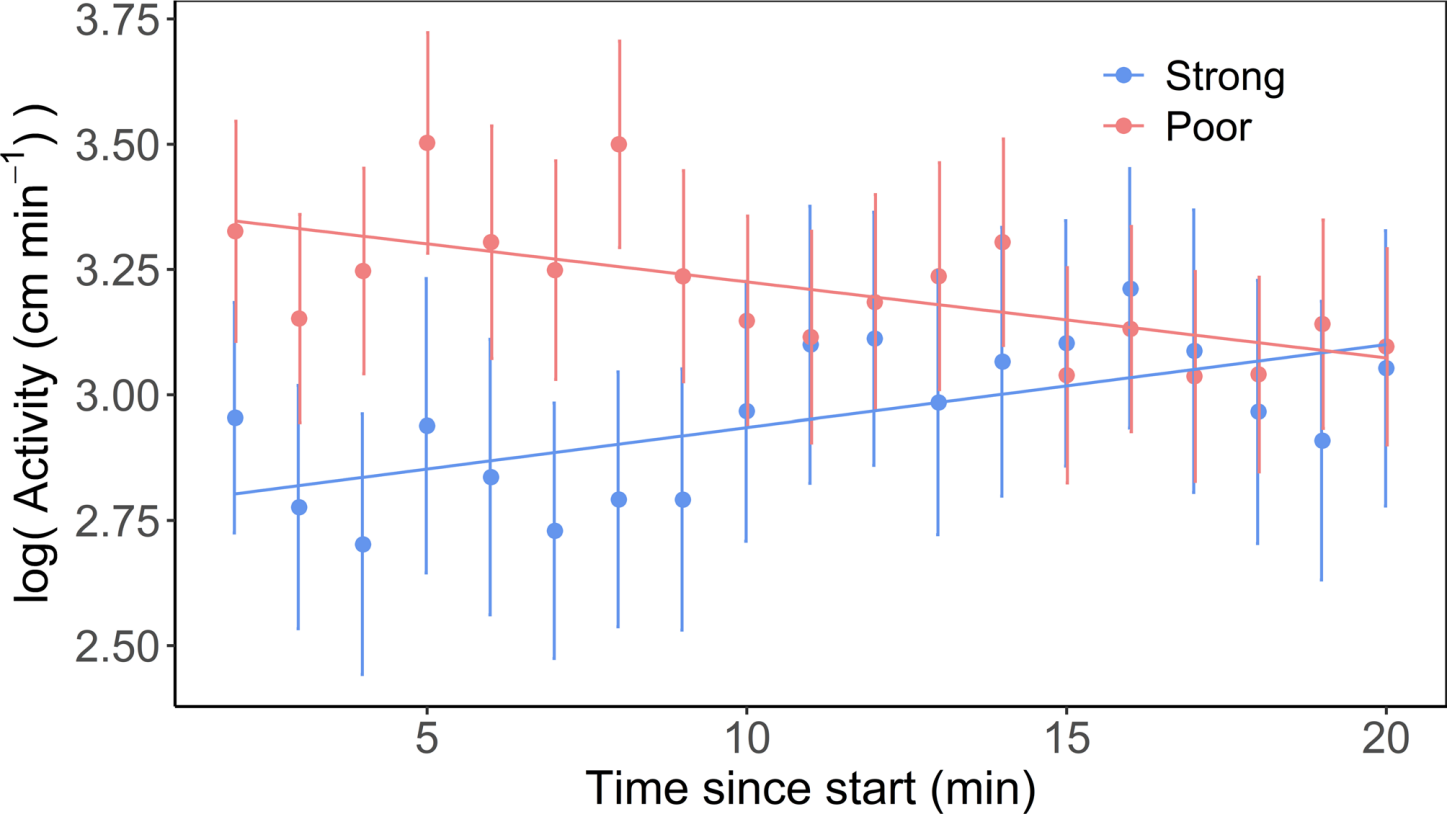
Locomotor response to a tank test, combining confinement and novelty, in salmon smolts classified as strong and poor swimmers (blue and red symbols, respectively). The solid lines represent predicted values based on the mixed-effects model (see Table S2), while the points indicate average values of observed data and its variability (standard error, SE).

Strong swimmers responded to the tank test with an average swimming speed ± S.E.M. of 40 ± 3 cm min^-1^ for strong swimmers and 41 ± 2 cm min^-1^ for poor swimmers. There were significant effects of swimmer type, time and the interaction between swimmer type and time on fish activity (LME, *p* < 0.05, Table S4). Activity of poor swimmers decreased through the course of the trials, while that of strong swimmers increased (Fig. 2). The variable test bout was initially included as a fixed effect but was removed because it was not statistically significant (*p* > 0.05) and its inclusion did not improve model fit (Table S4).

### Behavior and survival during migration

**Fig. 3:**
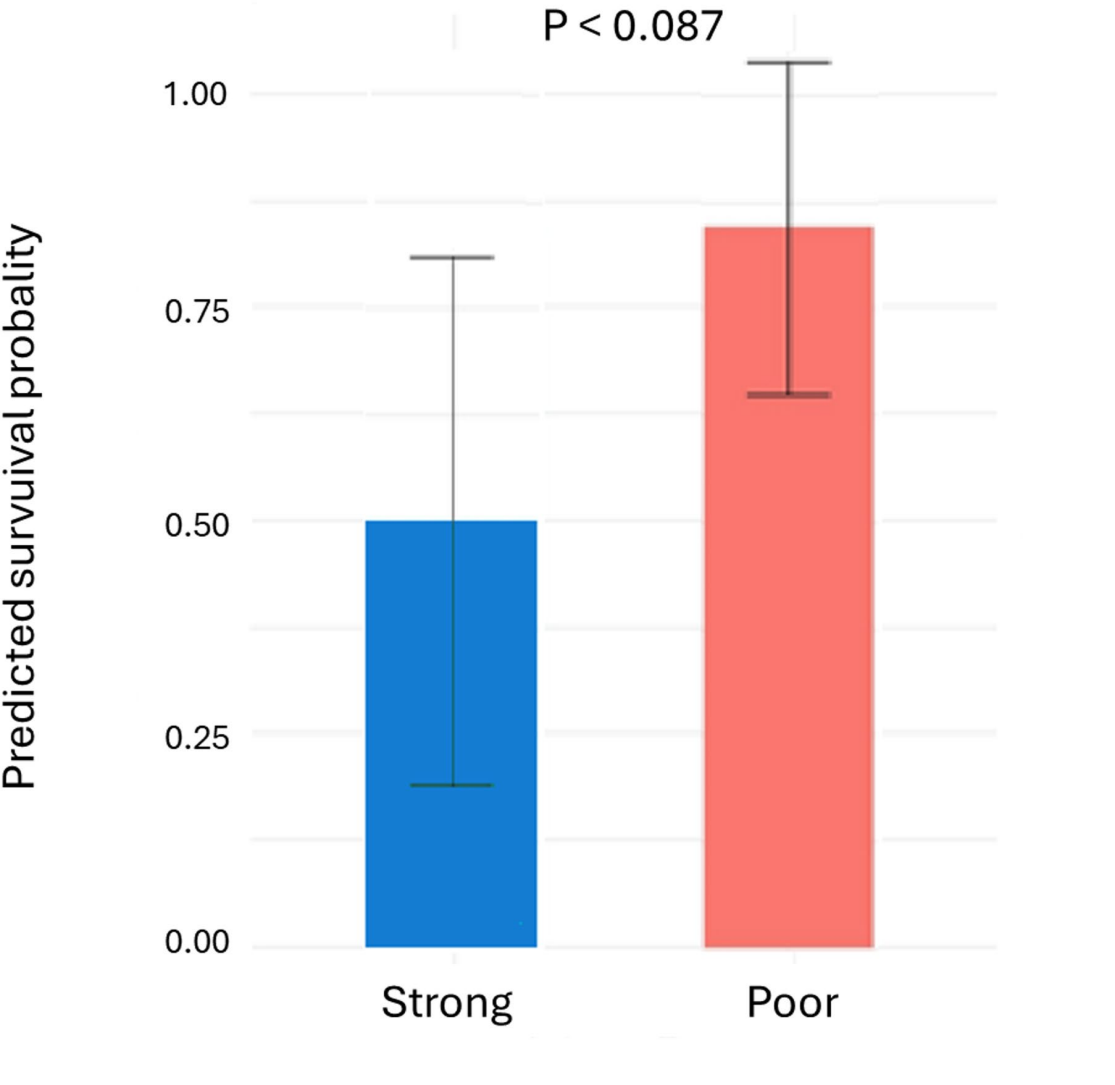
Predicted survival probability at the river mouth in salmon smolts classified as strong and poor swimmers. Smolts were released in the river stretch downstream a hydroelectric power plant 9 km from the river mouth. Values are mean ± 95% confidence. Parameter estimates and corresponding likelihood-ratio test statistics for the model fitted to predict survival probability are presented in the supplements (Table S4).

Of the 23 smolts released with acoustic tags, 16 individuals were detected at the river mouth receiver. The model estimated a survival probability of 0.50 (95% CI: 0.22–0.76) for the strong swimmers and 0.85 (95% CI: 0.55–0.96) for the poor swimmers. The model predicted a trend for different survival probability, which did not reach statistical significance (*p* = 0.087; Fig. 3).

The smolts used an average of 5.6 days (95% CI: 4.1–7.1 d) to migrate from Rygene to the river mouth (9 km). Strong swimmers averaged 4.6 days (95% CI: 2.0–7.3 d), while poor swimmers averaged 6.0 days (95% CI: 4.2–7.8 d). The effect of swim-type on migration time was not significant (*p* = 0.40).

Parameter estimates and corresponding likelihood-ratio test statistics for the model fitted to predict survival probability are presented in the supplements (Tables S5, S6).

## Discussion

The present study demonstrates behavioral differences in response to a tank test, combining novelty with confinement, among smolts classified as strong and poor swimmers by a forced swim test. Strong swimmers were less active at the beginning of the behavioral tank test but increased their activity over time, while poor swimmers showed the opposite trend. Furthermore, strong and poor swimmers also differed in their cortisone, but not cortisol, levels approximately 24 h after the swim test. No differences in heart morphology were detected between the groups of smolts with contrasting swimming capacities.

There were some discrepancies in the time to exhaustion observed across the forced swim test bouts. It is important to note that fish aggregated in low-flow areas near the inner wall of the tank were manually redirected into the main water flume using a dip net. The observed variation between bouts is most likely due to slight differences in hydraulic conditions between trials, as well as the effects of this manual intervention. Therefore, in this study, the classification of poor and strong swimmers was based on relative performance rather than absolute time to exhaustion. While the observed variation in exhaustion time between bouts may reflect true differences in swimming capacity, this cannot be conclusively determined. If such differences are genuine, they raise important questions about the link between swimming ability and the timing of smolt migration. Nevertheless, it is important to emphasize that variation between bouts did not significantly contribute to the model demonstrating behavioral differences between strong and poor swimmers. During the behavioral tank test, fish were moved to a tank that was relatively small compared to body size, exposing them to stress factors associated with confinement and an unfamiliar environment. When tested under similar conditions, activity level has been associated with the time taken to regain feeding in a novel environment and cortisol responsiveness to acute stress. Consequently, a low-activity response to confinement has been linked to a proactive coping style^14,20^, which includes a generally bolder BT and less accentuated cortisol responses to challenges than the reactive counterpart^12,20^. Alluding to this, Haraldstad et al.^17^ demonstrated that fish with low activity levels, when tested in identical tanks as in the current study, had a propensity to migrate through a turbine intake and suggested that these turbine migrators were bolder than their high-activity counterparts. The linear mixed-effects model applied in this study indicated that strong swimmers exhibited reduced activity during the tank test, with the effect most pronounced in the first 10 min. This finding aligns with previous research on selectively bred trout lines exhibiting contrasting stress coping styles. The trout line characterized by proactive behavioral traits showed lower initial locomotor activity during the first 10 min when tested under conditions similar to those of the present study^21^. The observed differences in locomotor activity—previously associated with boldness—support a potential relationship between aerobic capacity and BT in teleost fishes (e.g., Metcalfe et al.^10^). However, to further validate this relationship, future studies should consider reversing the experimental design—first characterizing individuals as bold or shy through behavioral testing and then assessing their swimming capacity. Such an approach would help clarify the directionality and robustness of the link between BT and swimming performance in migrating smolt.

As mentioned above, a proactive coping style, among other traits, is characterized by a less pronounced cortisol response to challenges, compared to reactive individuals^12^. In the present study, we did not detect any differences between poor and strong swimmers in plasma cortisol levels 24 h after the swim test. Still, poor swimmers responding with higher locomotor activity in the beginning of the tank test exhibited lower cortisone values compared to strong swimmers. Generally, cortisol is converted to its less active metabolite, cortisone, to protect against the detrimental effects of prolonged elevated cortisol levels^22^. Interestingly, studies in sea bass with contrasting cortisol responses to acute stress demonstrate an upregulation of the enzyme responsible for converting cortisol to cortisone (11β-hydroxysteroid dehydrogenase) in high-responding individuals^23^. Furthermore, in rainbow trout lines selected for high and low cortisol responses to acute stress, high cortisol responsiveness is accompanied by elevated cortisone levels^24^. In the present study, cortisone was not associated with high cortisol levels or with the total cortisone and cortisol concentrations. This suggests that the glucocorticoid differences between strong and poor swimmers are associated with the ability to convert cortisol to cortisone, rather than glucocorticoid production per se. Further studies are needed to investigate whether this difference is related to variations in glucocorticoid metabolism and 11β-hydroxysteroid dehydrogenase. Moreover, it is worth noting that cortisol levels in this study were generally elevated (≈600 ng/L) compared to typical values reported in Atlantic salmon, which often range between 0 and 200 ng/L (e.g. Madario et al.^25^). However, elevated cortisol levels are known to be associated with smoltification, and similar concentrations have been observed in smolts 24 h after transport^26^. Furthermore, it remains unclear whether the glucocorticoid differences observed in this study are a direct response to the swim trials or reflect pre-existing physiological states during smolt migration. Future studies should aim to incorporate baseline hormone measurements and explore minimally invasive sampling methods or glucocorticoid matrixes which are not as sensible to handling, such as feces cortisol^27^, to better capture the temporal dynamics of cortisol and cortisone during smolt migration.

In addition to behavioral traits, physiological performance is also likely to predict swimming capacity. Previous studies have linked swimming performance to cardiac morphology and function in farmed populations of rainbow trout^2^. Using a similar experimental design to the present study, Claireaux et al.^2^ showed that poor swimmers had rounded ventricles compared to strong swimmers, and this cardiac phenotype was associated with impaired cardiovascular performance. In contrast, we found no indication that cardiac size or shape impacted the swimming performance of migrating wild salmon smolts. This could suggest that cardiac shape does not restrict cardiac and physical performance in salmon at this life stage. Alternatively, none of the wild salmon smolts investigated in the present study had sufficiently rounded ventricles to affect their performance. In other words, variation in heart morphology in wild populations may not necessarily result in changes that influence swimming performance. Indeed, the rounded ventricular phenotype of farmed salmonids appears to be promoted by specific farming conditions characterized by elevated rearing temperatures and continuous light exposure^28,29^, which differ vastly from the environmental conditions experienced by salmon smolts in the wild.

In this study, acoustic telemetry demonstrated a non-significant trend towards higher survival rate among poor swimmers, despite the relatively small sample size of migrating smolts. However, this was not associated with the time taken to reach the sea. Several studies have indicated a connection between boldness and predator avoidance^30–32^. Notably, in this study, smolts were released downstream of a hydropower plant in a minimal discharge stretch, which provides suitable habitats for pike (*Esox lucius*), a well-known piscivorous predator in the river system. Considering that lower activity levels in similar tank tests, as conducted in the present study, have been associated with generally bolder BT^14,20,21^, it is tempting to suggest that the trend for a lower survival rate in strong swimmers could be related to a bolder BT. Still, it is important to note that post-tagging mortality was relatively high, with 2 of 15 strong swimmers and 5 of 15 poor swimmers dying before release. This raises the possibility of legacy effects from the behavioral testing and tagging procedures. Future studies should aim to minimize such impacts, for example by using less invasive tagging methods such as passive integrated transponders (PIT tags), which allow for larger sample sizes at lower cost. Including a control group of untested and unhandled fish would also help to better isolate the effects of experimental procedures on survival outcomes.

## Conclusions

This study highlights individual differences in swimming performance among Atlantic salmon smolts during their migration from natal rivers to ocean feeding grounds. These differences were linked to behavioral reactions to a combination of confinement and an unfamiliar environment, suggesting a relationship between swimming performance and bold-shy behavioral traits. Moreover, strong swimmers exhibited higher plasma cortisone, but not cortisol, 24 h after the swim test. Given that sustained elevated cortisol levels have been associated with hypertrophic cardiac growth, the lack of differences in plasma cortisol or heart morphology, between poor and strong swimmers might be related to a higher conversion of cortisol to cortisone in poor swimmers. This raises questions about the involvement of variations in glucocorticoid metabolism in swimming performance and behavior in salmon smolts. Additionally, we observed a tendency for lower survival rates during migration among strong swimmers, an effect likely related to increased predation. Further studies are needed to confirm these survival patterns and to better understand how predator pressure may influence the fitness consequences of swimming capacity, heart morphology, and boldness in migrating smolts. Given that no differences in heart morphology were detected between strong and poor swimmers, future research should investigate whether selection can act on the bold–shy continuum independently of cardiac morphology. Additionally, studies focusing on metabolic scope, cardiac output, and gill morphology are needed to explore a potential decoupling between cardiorespiratory performance and both swimming ability and behavioral traits in migrating salmon smolts.

## Supplementary Information

Supplementary Information 1.

Supplementary Information 2.

Supplementary Information 3.

Supplementary Information 4.

Supplementary Information 5.

## Data Availability

For data used in the statistical analysis see supplementary files to the manuscript.

## References

[1] Thorstad, E. et al. A critical life stage of the Atlantic salmon Salmo salar: Behaviour and survival during the smolt and initial post-smolt migration. *J. fish biol.* **81**, 500–542 (2012). 22803722 10.1111/j.1095-8649.2012.03370.x

[2] Claireaux, G. et al. Linking swimming performance, cardiac pumping ability and cardiac anatomy in rainbow trout. *J. Exp. Biol.* **208**, 1775–1784 (2005). 15879059 10.1242/jeb.01587

[3] Engdal, V. A. et al. State of the heart: Anatomical annotation and assessment of morphological cardiac variation in Atlantic salmon (Salmo salar L.). *Aquaculture* **578**, 740046 (2024).

[4] Johansen, I. B. et al. Bigger is not better: cortisol-induced cardiac growth and dysfunction in salmonids. *J. Exp. Biol.* **220**, 2545–2553 (2017). 28476893 10.1242/jeb.135046

[5] Culbert, B. M., Regish, A. M., Hall, D. J., McCormick, S. D. & Bernier, N. J. Neuroendocrine regulation of plasma cortisol levels during smoltification and seawater acclimation of Atlantic salmon. *Front. Endocrinol.* **13**, 859817 (2022). 10.3389/fendo.2022.859817 PMC9069684 35528002

[6] Kittilsen, S. et al. Melanin-based skin spots reflect stress responsiveness in salmonid fish. *Horm. Behav.* **56**, 292–298 (2009). 19539629 10.1016/j.yhbeh.2009.06.006

[7] Øverli, Ø., Winberg, S. & Pottinger, T. G. Behavioral and neuroendocrine correlates of selection for stress responsiveness in rainbow trout—a review. *Integr. Comp. Biol.* **45**, 463–474 (2005). 21676791 10.1093/icb/45.3.463

[8] Huntingford, F. et al. Coping strategies in a strongly schooling fish, the common carp Cyprinus carpio. *J. Fish Biol.* **76**, 1576–1591 (2010). 20557617 10.1111/j.1095-8649.2010.02582.x

[9] Killen, S. S. et al. Aerobic scope predicts dominance during early life in a tropical damselfish. *Func. Ecol.* **28**, 1367–1376 (2014).

[10] Metcalfe, N., Van Leeuwen, T. & Killen, S. Does individual variation in metabolic phenotype predict fish behaviour and performance?. *J. fish biol.* **88**, 298–321 (2016). 26577442 10.1111/jfb.12699 PMC4991269

[11] Rupia, E. J., Binning, S. A., Roche, D. G. & Lu, W. Fight-flight or freeze-hide? Personality and metabolic phenotype mediate physiological defence responses in flatfish. *J. Anim. Ecol.* **85**, 927–937 (2016). 27044558 10.1111/1365-2656.12524

[12] Koolhaas, J. et al. Coping styles in animals: Current status in behavior and stress-physiology. *Neurosci. Biobehav. Rev.* **23**, 925–935 (1999). 10580307 10.1016/s0149-7634(99)00026-3

[13] Øverli, Ø. et al. Evolutionary background for stress-coping styles: Relationships between physiological, behavioral, and cognitive traits in non-mammalian vertebrates. *Neurosci. Biobehav. Rev.* **31**, 396–412 (2007). 17182101 10.1016/j.neubiorev.2006.10.006

[14] Kittilsen, S., Ellis, T., Schjolden, J., Braastad, B. O. & Øverli, Ø. Determining stress-responsiveness in family groups of Atlantic salmon (Salmo salar) using non-invasive measures. *Aquaculture* **298**, 146–152 (2009).

[15] Dittman, A. H. & Quinn, T. P. Homing in Pacific salmon: Mechanisms and ecological basis. *J. of Exp. Biol.* **199**, 83–91 (1996). 9317381 10.1242/jeb.199.1.83

[16] Eliason, E. J. et al. Differences in thermal tolerance among sockeye salmon populations. *Science* **332**, 109–112 (2011). 21454790 10.1126/science.1199158

[17] Haraldstad, T., Haugen, T. O., Olsen, E. M., Forseth, T. & Höglund, E. Hydropower-induced selection of behavioural traits in Atlantic salmon (Salmo salar). *Sci. Rep.* **11**, 16444 (2021). 34385548 10.1038/s41598-021-95952-1 PMC8360942

[18] Schindelin, J. et al. Fiji: an open-source platform for biological-image analysis. *Nat. Methods* **9**, 676–682 (2012). 22743772 10.1038/nmeth.2019 PMC3855844

[19] R Core Team (2022). R: A language and environment for statistical computing. R Foundation for Statistical Computing, Vienna, Austria. URL https://www.R-project.org/.

[20] Øverli, Ø., Sørensen, C. & Nilsson, G. E. Behavioral indicators of stress-coping style in rainbow trout: Do males and females react differently to novelty?. *Physiol. Behav.* **87**, 506–512 (2006). 16455115 10.1016/j.physbeh.2005.11.012

[21] Backström, T., Schjolden, J., Øverli, Ø., Thörnqvist, P. O. & Winberg, S. Stress effects on AVT and CRF systems in two strains of rainbow trout (Oncorhynchus mykiss) divergent in stress responsiveness. *Hormon. Behav.* **59**, 180–186 (2011). 10.1016/j.yhbeh.2010.11.008 21087609

[22] Tomlinson, J. W. & Stewart, P. M. Cortisol metabolism and the role of 11β-hydroxysteroid dehydrogenase. *Best Practi. Res. Cl.* **15**, 61–78 (2001). 10.1053/beem.2000.0119 11469811

[23] Samaras, A. & Pavlidis, M. Regulation of divergent cortisol responsiveness in European sea bass, Dicentrarchus labrax L.. *PLoS ONE* **13**, e0202195 (2018). 30096195 10.1371/journal.pone.0202195 PMC6086447

[24] Pottinger, T. & Moran, T. Differences in plasma cortisol and cortisone dynamics during stress in two strains of rainbow trout (Oncorhynchus mykiss). *J. fish biol.* **43**, 121–130 (1993).

[25] Madaro, A. et al. Acute stress response on Atlantic salmon: A time-course study of the effects on plasma metabolites, mucus cortisol levels, and head kidney transcriptome profile. *Fish Physiol Biochem* **49**, 97–116 (2023). 36574113 10.1007/s10695-022-01163-4 PMC9935726

[26] Culbert, B. M., Regish, A. M., Hall, D. J., McCormick, S. D. & Bernier, N. J. Neuroendocrine regulation of plasma cortisol levels during smoltification and seawater acclimation of Atlantic salmon. *Front in Endocrinol* **13**, 859817 (2022). 10.3389/fendo.2022.859817 PMC9069684 35528002

[27] Ding, J. W. et al. Comparative assessment of plasma cortisol and fecal corticoid metabolites (FCM) of Atlantic salmon (Salmo salar L.) subjected to acute-and long-term stress. *Aquaculture* **568**, 739299 (2023).

[28] Frisk, M. et al. Intensive smolt production is associated with deviating cardiac morphology in Atlantic salmon (Salmo salar L.). *Aquaculture* **529**, 735615 (2020).

[29] Vindas, M. A. et al. Importance of environmental signals for cardiac morphological development in Atlantic salmon. *J Exp. Biol.* **227** (2024). 10.1242/jeb.247557 PMC11529873 39387107

[30] Brown, G. E., Elvidge, C. K., Ramnarine, I., Chivers, D. P. & Ferrari, M. C. Personality and the response to predation risk: Effects of information quantity and quality. *Anim. Cogn.* **17**, 1063–1069 (2014). 24563039 10.1007/s10071-014-0738-z

[31] Ehlman, S. M. et al. Intermediate turbidity elicits the greatest antipredator response and generates repeatable behaviour in mosquitofish. *Anim Behav.* **158**, 101–108 (2019).

[32] Garamszegi, L. Z., Markó, G. & Herczeg, G. A meta-analysis of correlated behaviors with implications for behavioral syndromes: Relationships between particular behavioral traits. *Behav. Ecol.* **24**, 1068–1080 (2013).

