# Supplementary material for: Exploring the links between swimming performance, glucocorticoid profiles, behavioral types and cardiac morphology in migrating Atlantic salmon (Salmo salar) smolts
Source: Sci Rep. 2026 Jan 16;16:5560. doi: 10.1038/s41598-026-35402-y (PMC12891565; doi:10.1038/s41598-026-35402-y)
Supplement: Supplementary file 5 — Supplementary Information 5. [file 41598_2026_35402_MOESM5_ESM.docx]

**Supplementary material**

**S1. Steroid hormones analysis in small volumes of Atlantic salmon plasma by LC-MS/MS**

A sensitive reverse phase- liquid chromatography-tandem mass spectrometry (LC-MS/MS) analysis method for the identification and quantification of multiple endogenous steroid hormones in small volumes (5-20µL) of Atlantic salmon (*Salmo salar*) plasma.

Cortisol and cortisone were selected as anlytes.

**Chemicals and reagents:**

All mentioned steroid compounds and their corresponding stable isotope-labeled standards (100 µg/mL in methanol stock solutions of: cortisol -13C3, cortisone-d8,17-hydroxyprogesterone-d8,11-deoxycortisol-d5,13C3-androstenedione and 13C3-testosterone) were of analytical or CRM (certified reference material) grade and provided from Merck-Sigma (Darmstadt, Germany). Ultrapure water (18.2Ω, TOC<1) was produced by Elga Ultrapure Laboratory water purification system (Lane End, UK).

The solvents and reagents (acetonitrile, methanol, formic acid, hexane, ethyl acetate) were HPLC, LC-MS or analytical grade and supplied from VWR International (Radnor, US).

Individual stock solutions of 1mg/mL of each steroid were prepared in methanol and stored at -20⁰C. Working solutions were freshly prepared by dilution in 50: 50water: methanol (v/v) to 0.01, 0.1,1 and 10 µg/mL.

The internal standard mixture used to spike each sample, of 100ng/mL of cortisol -13C3, cortisone-d8 was freshly prepared in 90:10 water: methanol(v/v) before each analysis.

**Sample preparation**

Samples were collected in 2-ml Eppendorf tubes and stored at

-80⁰C until analysis.

The samples were thawed on ice, then 10µL were transferred to a new 2 mL Precellys homogenization tube prefilled with 1.4 mm zirconium oxide beads (VWR, Radnor, US). An aliquot of 10µL internal standard mix was added to each sample, followed by dilution with 200µL pure water and sonication for 5 minutes (on ice and water blend) in an ultrasound bath (VWR, Radnor, US).

The homogenization step was performed thrice on a Precellys 24 evolution tissue homogenizer (Bertin Technologies, Montigny-le-Bretonneux, France). The entire sample mixture was loaded onto 1 mL Novum solid liquid extraction (SLE) 30mg sorbent cartridge (Phenomenex, Torrance, US) by using a Supelco vacuum manifold (Merck, Darmstadt, Germany). 5” Hg vacuum was applied for 10 seconds, then 5 minutes waiting time for gravity elution.

The compounds of interest were eluted twice with 600µL of 75:25 ethyl acetate: hexane (v/v).

The extracts were then evaporated under a gentle stream of nitrogen on 37⁰C heating block by using a Reacti-Vap Evaporator (Thermo Fisher Scientific, Waltham, US).The residue was redissolved in 100µL of 50:50 water :methanol (v/v), filtered with Spin-X centrifuge tube filter, 0.22µm (Costar, Washington D.C., US) and transferred to a HPLC vial with insert (Agilent, Santa Clara, US).

**Instrumentation and analytical conditions (LC-MS/MS)**

Method optimization and analysis of steroid hormones in salmon plasma were conducted on LC-ESI-MS/MS (liquid chromatography- electrospray ionization-tandem mass spectrometry) setup consisting of binary pump, degasser and autosampler with thermostat (Agilent Technologies, Santa Clara, US) coupled to an API 4000 triple- quadrupole mass spectrometer (SCIEX, Toronto, Canada) equipped with Turbo Ion Spray.

The temperature of the autosampler was set at 5⁰C.

The chromatographic separation was carried- out on a reversed phase Kinetex-F5 column, 100x2.1 mm, 2.5µm core shell particles (Phenomenex, Torrance, US).

The column temperature was 35⁰C.

The mobile phase consisted of 0.1% formic acid in water (A) and 0.1% formic acid in acetonitrile (B). The flow rate was 0.25mL/min and the injection volume 40µL.

The separated compounds were detected in negative and positive ionization - multiple reaction monitoring (MRM) mode in the same run analysis (20 minutes total analysis time), selecting one precursor ion to two products ion transitions (a quantifier and a qualifier) for each compound. (Table S1)

The quantifier ion is used to quantify the analyte, by using the area of the extract ion chromatogram (XIC) after peak integration. It’s used to be the most abundant ion in the mass spectrum of the target compound. This area is used to calculate the concentration after calibration.

The qualifier ion is used to identify the analyte. The ion ratio of the quantifier and qualifier gives evidence about the correct identity of the species being monitored.

**Table S1.** The mass transitions and retention times of the compounds and their respectively isotope labelled internal standards**:**

| **Compound** | **Ion mode** | **MRM transitions (m/z)** | **Retention time(min)** |
| --- | --- | --- | --- |
| Cortisol | ESI negative | 407.1>331*,297 | 8.52 |
| 13C3-Cortisol | ESI negative | 410.2>334,300 | 8.52 |
| Cortisone | ESI negative | 405.5>329*,301 | 8.8 |
| Cortisone-d8 | ESI negative | 413.4>337,309 | 8.8 |

m/z = mass to charge ratio; *quantifier ion

The software used for controlling this equipment, acquiring, and processing the data was Analyst Version 1.7 (SCIEX, Toronto, Canada).

The following validation parameters: specificity, detection limit (LoD), lower limit of quantification limit (LLoQ), linearity, precision, accuracy, recovery, and matrix effects were assessed by spiking salmon plasma with the corresponding isotope labelled standards analogs of each compound as surrogate standard.

The specificity was checked by analyzing diverse kinds of salmon plasma. No interfering peaks could be detected at the respectively retention time of each steroid. Matrix effects were evaluated by comparing data from calibration curves in diluent to matrix-matched ones at different ranges for each compound.

External standard calibration curves and stable isotope labelled internal standards are used for the quantitative assay.

The calibration standards were prepared in dilution solution (50:50 water: methanol (v/v)) as surrogate matrix based on the correction factors calculated for each compound related to their respectively matrix effects and recovery values.

The response of each analyte was linear and gave a correlation coefficient of R²≥0.99. The concentration in each sample was calculated by using the peak area ratio of the analytes to internal standards versus the nominal concentration ratio (analyte to internal standard) and linear regression analysis. When lower or higher amounts of plasma samples were used to analysis the volume of the intern standard mixture added to each sample was adapted accordingly.

Two quality control (QC) samples prepared in salmon plasma by spiking the matrix with a known concentration of steroid standards and internal standards were used for each run to estimate the **inter-assay precision** (0.39≤RSD% ≤6.96) **and accuracy** (94 to 109%) of the analytical method.

**Intra-assay precision and accuracy** (95 to 118%) for this method were calculated using six replicates of the control quality (QC) samples spiked at the same level analyzed during a single analytical run(6.3≤RSD%≤10).

Precision (should be less than 15%) was expressed as the percentage relative standard deviations (RSD%) of the control samples spiked at same level. Accuracy was expressed as the ratio of calculated concentration to known concentration (100±20%).

The extraction recoveries were between 53 and 90% for all analytes.

LOD (limit of detection) was based on 3xS/N (signal to noise) ratio and LLOQ (lower limit of quantification) was estimated as the lowest concentration point of the calibration curves used in the validation study. (Table S2)

**Table S2**. Summary of LOD, LLOQ calculated by spiking of salmon plasma with the corresponding isotope labelled standard.

| **Analyte** | **LOD (ng/mL)** | **LLOQ (ng/mL)** |
| --- | --- | --- |
| Cortisol | 0.08 | 0.25 |
| Cortisone | 0.08 | 0.25 |

**Table S3.** Summary of statistical results from the t-student’s test for cardiac morphology metrics in strong and poor swimmers for Atlantic salmon smolts. Abbreviations: df = degrees of freedom.

| **Cardiac morphology** | **t-value** | **df** | **p-value** |
| --- | --- | --- | --- |
| Cardiosomatic index (CSI) | -0.23 | 28 | 0.82 |
| Relative ventricule mass (RVM) | 0.63 | 19 | 0.53 |
| Ventricular height : width ratio | -0.17 | 31 | 0.86 |
| Bulbus weight : ventricular width ratio | 0.56 | 28 | 0.58 |
| Ventricular bulbus angle | 0.36 | 34 | 0.72 |
| Ventricular asymmetry angle | 0.27 | 31 | 0.79 |

**Table S4.** Summary statistics of best-fit linear mixed model on the effects of behavioral test duration (time) and swimmer type (strong vs poor) on locomotion activity of salmon smolts. The variable trial was initially included as a fixed effect but was removed because it was not statistically significant (p > 0.05) and its inclusion did not improve model fit (model without trial: AIC = 1759.79; model with trial: AIC = 1762.97; ΔAIC = 3.18).

| **Fixed effects** | | | | | |
| --- | --- | --- | --- | --- | --- |
|  | Value | SE | DF | t-value | p-value |
| Intercept | 2.770 | 0.213 | 790 | 13.026 | 0.000 |
| Swimmer type (poor) | 0.607 | 0.294 | 42 | 2.064 | 0.045 |
| Time | 0.016 | 0.006 | 790 | 2.927 | 0.003 |
| Swimmer type (poor): time | -0.031 | 0.008 | 790 | -4.061 | 0.001 |
|  | | | | | |
| **Random effects** | | | | | |
|  | Intercept | Residual |  |  |  |
| Standard Deviation | 0.921 | 0.617 |  |  |  |

**Table S5.** Parameter estimates and corresponding likelihood-ratio test statistics for the model fitted to predict survival probability of Atlantic salmon smolts to the Nidelva river mouth for strong and poor swimmers.

| Parameter estimates | | | |
| --- | --- | --- | --- |
| Term | Coeff. | SE | P |
| Intercept | 1.705 | 0.77 | 0.026 |
| Strong swimmers | -1.705 | 0.99 | 0.087 |

**Table S6.** Parameter estimates and corresponding likelihood-ratio test statistics for the model fitted to predict the migration speed of Atlantic salmon smolts to the river mouth of Nidelva for strong and poor swimmers.

| Parameter estimates | | | |
| --- | --- | --- | --- |
| Term | Coeff. | SE | P |
| Intercept | 6.032 | 0.91 | <0.001 |
| Strong swimmers | -1.409 | 1.63 | 0.402 |
